# Bis(1*H*-imidazole-κ*N* ^3^)bis­(2-oxidopyridinium-3-carboxyl­ato-κ^2^ *O* ^2^,*O* ^3^)cobalt(II)

**DOI:** 10.1107/S1600536809028694

**Published:** 2009-07-25

**Authors:** Bing-Yu Zhang, Jing-Jing Nie, Duan-Jun Xu

**Affiliations:** a Department of Chemistry, Zhejiang University, Hangzhou 310027, People’s Republic of China

## Abstract

In the mol­ecule of the title Co^II^ complex, [Co(C_6_H_4_NO_3_)_2_(C_3_H_4_N_2_)_2_], the Co^II^ atom is located on a twofold rotation axis and chelated by two oxidopyridiniumcarboxyl­ate anions and further *cis*-coordinated by two imidazole ligands in a distorted octa­hedral geometry. The shorter C—O bond distance of 1.260 (2) Å suggests electron delocalization between the oxido group and the pyridinium ring. The uncoordinated carboxyl­ate O atom links with the imidazole and pyridinium rings of adjacent mol­ecules *via* N—H⋯O hydrogen bonding. Weak C—H⋯O hydrogen bonding is also present in the crystal structure.

## Related literature

For the isostructural Ni^II^ complex, see: Zhang *et al.* (2009 ▶). For the shorter C—O bond distance between the pyridine ring and the hydr­oxy-O atom in 2-oxidopyridinium-3-carboxyl­ate complexes and in 2-hydroxy­pyridine­carboxyl­ate complexes, see: Yao *et al.* (2004 ▶); Yan & Hu (2007*a* ▶,*b* ▶); Wen & Liu (2007 ▶); Quintal *et al.* (2002 ▶). For the corresponding C—O bond distances in 2-hydroxy­benzencarboxylic acid and in metal complexes of 2-hydroxy­benzencarboxyl­ate, see: Munshi & Guru Row (2006 ▶); Su & Xu (2005 ▶); Li *et al.* (2005 ▶).
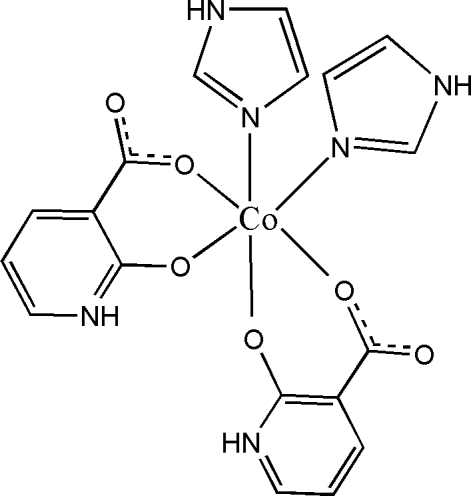


## Experimental

### 

#### Crystal data

[Co(C_6_H_4_NO_3_)_2_(C_3_H_4_N_2_)_2_]
*M* *_r_* = 471.30
Monoclinic, 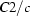

*a* = 16.594 (2) Å
*b* = 10.0524 (12) Å
*c* = 12.8271 (15) Å
β = 111.407 (4)°
*V* = 1992.1 (4) Å^3^
*Z* = 4
Mo *K*α radiation
μ = 0.91 mm^−1^
*T* = 294 K
0.40 × 0.30 × 0.26 mm

#### Data collection

Rigaku R-AXIS RAPID IP diffractometer
Absorption correction: multi-scan (*ABSCOR*; Higashi, 1995 ▶) *T*_min_ = 0.665, *T*_max_ = 0.790
10627 measured reflections
1824 independent reflections
1527 reflections with *I* > 2σ(*I*)
*R*_int_ = 0.034

#### Refinement

*R*[*F* ^2^ > 2σ(*F* ^2^)] = 0.028
*wR*(*F* ^2^) = 0.065
*S* = 1.07
1824 reflections
141 parameters
H-atom parameters constrained
Δρ_max_ = 0.24 e Å^−3^
Δρ_min_ = −0.22 e Å^−3^

### 

Data collection: *PROCESS-AUTO* (Rigaku, 1998 ▶); cell refinement: *PROCESS-AUTO*; data reduction: *CrystalStructure* (Rigaku/MSC, 2002 ▶); program(s) used to solve structure: *SIR92* (Altomare *et al.*, 1993 ▶); program(s) used to refine structure: *SHELXL97* (Sheldrick, 2008 ▶); molecular graphics: *ORTEP-3* (Farrugia, 1997 ▶); software used to prepare material for publication: *WinGX* (Farrugia, 1999 ▶).

## Supplementary Material

Crystal structure: contains datablocks I, global. DOI: 10.1107/S1600536809028694/hk2743sup1.cif

Structure factors: contains datablocks I. DOI: 10.1107/S1600536809028694/hk2743Isup2.hkl

Additional supplementary materials:  crystallographic information; 3D view; checkCIF report

## Figures and Tables

**Table 1:** Selected bond lengths (Å)

Co—O1	2.0684 (13)
Co—O3	2.1402 (13)
Co—N2	2.1107 (16)

**Table 2:** Hydrogen-bond geometry (Å, °)

*D*—H⋯*A*	*D*—H	H⋯*A*	*D*⋯*A*	*D*—H⋯*A*
N1—H1⋯O2^i^	0.86	1.93	2.789 (2)	177
N3—H3⋯O2^ii^	0.86	2.04	2.806 (2)	148
C3—H3*A*⋯O3^iii^	0.93	2.43	3.341 (3)	168

Symmetry codes: (i) 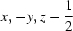; (ii) 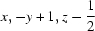; (iii) 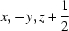.

**Fig. 1.**
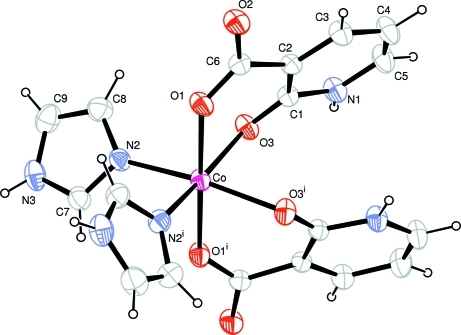
The molecular structure of the title complex with 40% probability displacement ellipsoids (arbitrary spheres for H atoms) [symmetry code: (i) 1 - x, y, 1/2 - z].

## supplementary crystallographic information

### Comment

The title Co^II^ complex is isostructural with the Ni^II^ complex (Zhang *et al.* 2009).

In the title molecule, the Co atom is located on a twofold axis and is coordinated by two imidazole molecules in *cis*-configuration, two oxidopyridinium-carboxylate anions further chelate to the Co atom with carboxyl-O and deprotonated hydroxy-O atoms to complete the distorted octahedral coordination geometry (Fig. 1 and Table 1). The benzene ring is twisted with respect to the carboxyl group and O1/O3/Co coordination plane with dihedral angles of 21.52 (13)° and 41.05 (7)°, respectively. The shorter C1—O3 [1.260 (2) Å] bond agrees with those found in the isostructural Ni complex (Zhang *et al.* 2009) and in the other transition metal complexes of oxidopyridinium-carboxylate (Yao *et al.*, 2004; Yan & Hu, 2007*a*, b; Wen & Liu, 2007), it is also consistent with that found in hydroxy-pyridinecarboxylate complexes (Quintal *et al.* 2002). This finding suggests the electron delocalization between pyridine ring and hydroxy group. But this shorter C1—O3 bond is much different from the C—O bond distance of *ca* 1.35 Å between benzene ring and hydroxy-O atom found in hydroxybenzencarboxylic acid (Munshi & Row, 2006) and metal complexes of hydroxybenzenecarboxylate (Su & Xu, 2005; Li *et al.*, 2005).

The uncoordinated carboxyl O atom simultaneously links with the imidazole and pyridinium rings *via* N—H···O hydrogen bonding of adjacent molecules (Table 2). Weak C—H···O hydrogen bonding is also present in the crystal structure.

### Experimental

2-Hydroxy-pyridine-3-carboxylic acid (0.13 g, 1 mmol), NaOH (0.04 g, 1 mmol), imidazole (0.14 g, 2 mmol) and CoCl_2_.6H_2_O (0.24 g, 1 mmol) were dissolved in water (15 ml). The solution was refluxed for 4.5 h, after cooling to room temperature the solution was filtered. The single crystals of the title complex were obtained from the filtrate after one week.

### Refinement

H atoms were placed in calculated positions with C—H = 0.93 and N—H = 0.86 Å, and refined in riding mode with *U*_iso_(H) = 1.2*U*_eq_(C,*N*).

### Crystal data

**Table:**

[Co(C_6_H_4_NO_3_)_2_(C_3_H_4_N_2_)_2_]	*F*(000) = 964
*M**_r_* = 471.30	*D*_x_ = 1.571 Mg m^−^^3^
Monoclinic, *C*2/*c*	Mo *K*α radiation, λ = 0.71073 Å
Hall symbol: -C 2yc	Cell parameters from 4226 reflections
*a* = 16.594 (2) Å	θ = 2.5–25.2°
*b* = 10.0524 (12) Å	µ = 0.91 mm^−^^1^
*c* = 12.8271 (15) Å	*T* = 294 K
β = 111.407 (4)°	Block, pink
*V* = 1992.1 (4) Å^3^	0.40 × 0.30 × 0.26 mm
*Z* = 4	

### Data collection

**Table:**

Rigaku R-AXIS RAPID IP diffractometer	1824 independent reflections
Radiation source: fine-focus sealed tube	1527 reflections with *I* > 2σ(*I*)
graphite	*R*_int_ = 0.034
Detector resolution: 10.00 pixels mm^-1^	θ_max_ = 25.4°, θ_min_ = 2.4°
ω scans	*h* = −20→20
Absorption correction: multi-scan (*ABSCOR*; Higashi, 1995)	*k* = −12→12
*T*_min_ = 0.665, *T*_max_ = 0.790	*l* = −14→15
10627 measured reflections	

### Refinement

**Table:**

Refinement on *F*^2^	Primary atom site location: structure-invariant direct methods
Least-squares matrix: full	Secondary atom site location: difference Fourier map
*R*[*F*^2^ > 2σ(*F*^2^)] = 0.028	Hydrogen site location: inferred from neighbouring sites
*wR*(*F*^2^) = 0.065	H-atom parameters constrained
*S* = 1.07	*w* = 1/[σ^2^(*F*_o_^2^) + (0.0231*P*)^2^ + 1.9358*P*] where *P* = (*F*_o_^2^ + 2*F*_c_^2^)/3
1824 reflections	(Δ/σ)_max_ < 0.001
141 parameters	Δρ_max_ = 0.24 e Å^−^^3^
0 restraints	Δρ_min_ = −0.22 e Å^−^^3^

### Special details

**Table:**

Geometry. All e.s.d.'s (except the e.s.d. in the dihedral angle between two l.s. planes) are estimated using the full covariance matrix. The cell e.s.d.'s are taken into account individually in the estimation of e.s.d.'s in distances, angles and torsion angles; correlations between e.s.d.'s in cell parameters are only used when they are defined by crystal symmetry. An approximate (isotropic) treatment of cell e.s.d.'s is used for estimating e.s.d.'s involving l.s. planes.
Refinement. Refinement of *F*^2^ against ALL reflections. The weighted *R*-factor *wR* and goodness of fit *S* are based on *F*^2^, conventional *R*-factors *R* are based on *F*, with *F* set to zero for negative *F*^2^. The threshold expression of *F*^2^ > σ(*F*^2^) is used only for calculating *R*-factors(gt) *etc*. and is not relevant to the choice of reflections for refinement. *R*-factors based on *F*^2^ are statistically about twice as large as those based on *F*, and *R*- factors based on ALL data will be even larger.

### Fractional atomic coordinates and isotropic or equivalent isotropic displacement parameters (Å^2^)

**Table:**

	*x*	*y*	*z*	*U*_iso_*/*U*_eq_	
Co	0.5000	0.24771 (3)	0.2500	0.02958 (13)	
N1	0.61270 (11)	−0.12644 (16)	0.30331 (13)	0.0369 (4)	
H1	0.6138	−0.1391	0.2376	0.044*	
N2	0.58362 (11)	0.39282 (16)	0.22623 (13)	0.0348 (4)	
N3	0.62322 (13)	0.56271 (17)	0.14899 (16)	0.0468 (5)	
H3	0.6198	0.6308	0.1070	0.056*	
O1	0.56130 (10)	0.24739 (13)	0.42230 (11)	0.0392 (4)	
O2	0.62213 (10)	0.16732 (14)	0.59300 (10)	0.0410 (4)	
O3	0.58952 (9)	0.09062 (13)	0.25972 (10)	0.0352 (3)	
C1	0.59989 (12)	0.00073 (18)	0.33196 (15)	0.0293 (4)	
C2	0.60117 (12)	0.01700 (18)	0.44393 (15)	0.0288 (4)	
C3	0.61152 (14)	−0.0923 (2)	0.51100 (17)	0.0375 (5)	
H3A	0.6115	−0.0814	0.5830	0.045*	
C4	0.62211 (16)	−0.2203 (2)	0.47472 (18)	0.0454 (6)	
H4	0.6278	−0.2938	0.5209	0.055*	
C5	0.62375 (16)	−0.2340 (2)	0.37099 (19)	0.0449 (6)	
H5	0.6325	−0.3175	0.3457	0.054*	
C6	0.59387 (12)	0.15307 (19)	0.48875 (15)	0.0299 (4)	
C7	0.55679 (15)	0.4904 (2)	0.15344 (18)	0.0396 (5)	
H7	0.4990	0.5071	0.1105	0.048*	
C8	0.67219 (15)	0.4046 (2)	0.27010 (19)	0.0458 (6)	
H8	0.7094	0.3488	0.3242	0.055*	
C9	0.69703 (16)	0.5089 (2)	0.2231 (2)	0.0510 (6)	
H9	0.7533	0.5381	0.2383	0.061*	

### Atomic displacement parameters (Å^2^)

**Table:**

	*U*^11^	*U*^22^	*U*^33^	*U*^12^	*U*^13^	*U*^23^
Co	0.0402 (2)	0.02722 (19)	0.0222 (2)	0.000	0.01241 (16)	0.000
N1	0.0554 (12)	0.0342 (9)	0.0257 (9)	0.0042 (8)	0.0204 (8)	−0.0020 (7)
N2	0.0400 (11)	0.0327 (9)	0.0318 (9)	0.0006 (8)	0.0132 (8)	0.0052 (7)
N3	0.0641 (14)	0.0341 (10)	0.0528 (12)	−0.0039 (9)	0.0340 (11)	0.0065 (9)
O1	0.0589 (10)	0.0309 (7)	0.0245 (7)	0.0071 (7)	0.0115 (7)	−0.0005 (6)
O2	0.0612 (10)	0.0410 (8)	0.0201 (7)	0.0046 (7)	0.0142 (7)	−0.0032 (6)
O3	0.0529 (9)	0.0334 (7)	0.0254 (7)	0.0071 (6)	0.0214 (7)	0.0040 (6)
C1	0.0330 (11)	0.0313 (10)	0.0254 (10)	0.0008 (8)	0.0129 (8)	−0.0023 (8)
C2	0.0332 (11)	0.0319 (10)	0.0227 (10)	0.0011 (8)	0.0118 (8)	−0.0005 (8)
C3	0.0507 (14)	0.0394 (11)	0.0271 (10)	0.0019 (10)	0.0196 (10)	0.0012 (9)
C4	0.0715 (17)	0.0337 (11)	0.0357 (12)	0.0057 (11)	0.0250 (12)	0.0072 (9)
C5	0.0686 (16)	0.0295 (11)	0.0415 (13)	0.0051 (10)	0.0259 (12)	0.0002 (9)
C6	0.0332 (11)	0.0354 (10)	0.0243 (10)	−0.0003 (9)	0.0142 (8)	−0.0014 (8)
C7	0.0446 (13)	0.0367 (11)	0.0375 (12)	0.0002 (10)	0.0149 (10)	0.0041 (9)
C8	0.0411 (14)	0.0475 (13)	0.0471 (14)	0.0035 (10)	0.0141 (11)	0.0018 (10)
C9	0.0435 (15)	0.0493 (14)	0.0666 (17)	−0.0068 (11)	0.0278 (13)	−0.0092 (12)

### Geometric parameters (Å, °)

**Table:**

Co—O1^i^	2.0684 (13)	O2—C6	1.253 (2)
Co—O1	2.0684 (13)	O3—C1	1.260 (2)
Co—O3^i^	2.1402 (13)	C1—C2	1.438 (3)
Co—O3	2.1402 (13)	C2—C3	1.367 (3)
Co—N2	2.1107 (16)	C2—C6	1.506 (3)
Co—N2^i^	2.1107 (16)	C3—C4	1.401 (3)
N1—C5	1.357 (3)	C3—H3A	0.9300
N1—C1	1.368 (2)	C4—C5	1.348 (3)
N1—H1	0.8600	C4—H4	0.9300
N2—C7	1.315 (2)	C5—H5	0.9300
N2—C8	1.374 (3)	C7—H7	0.9300
N3—C7	1.339 (3)	C8—C9	1.347 (3)
N3—C9	1.359 (3)	C8—H8	0.9300
N3—H3	0.8600	C9—H9	0.9300
O1—C6	1.258 (2)		
			
O1^i^—Co—O1	179.82 (7)	O3—C1—C2	126.95 (17)
O1^i^—Co—N2	86.54 (6)	N1—C1—C2	115.31 (16)
O1—Co—N2	93.59 (6)	C3—C2—C1	119.33 (17)
O1^i^—Co—N2^i^	93.59 (6)	C3—C2—C6	119.94 (16)
O1—Co—N2^i^	86.54 (6)	C1—C2—C6	120.71 (16)
N2—Co—N2^i^	92.57 (9)	C2—C3—C4	122.10 (18)
O1^i^—Co—O3^i^	82.90 (5)	C2—C3—H3A	119.0
O1—Co—O3^i^	96.97 (5)	C4—C3—H3A	119.0
N2—Co—O3^i^	168.64 (5)	C5—C4—C3	118.14 (19)
N2^i^—Co—O3^i^	92.26 (6)	C5—C4—H4	120.9
O1^i^—Co—O3	96.97 (5)	C3—C4—H4	120.9
O1—Co—O3	82.90 (5)	C4—C5—N1	120.26 (19)
N2—Co—O3	92.26 (6)	C4—C5—H5	119.9
N2^i^—Co—O3	168.64 (5)	N1—C5—H5	119.9
O3^i^—Co—O3	84.91 (8)	O2—C6—O1	122.54 (17)
C5—N1—C1	124.81 (17)	O2—C6—C2	117.41 (17)
C5—N1—H1	117.6	O1—C6—C2	120.05 (16)
C1—N1—H1	117.6	N2—C7—N3	111.4 (2)
C7—N2—C8	105.13 (18)	N2—C7—H7	124.3
C7—N2—Co	123.24 (15)	N3—C7—H7	124.3
C8—N2—Co	131.44 (14)	C9—C8—N2	109.9 (2)
C7—N3—C9	107.46 (18)	C9—C8—H8	125.1
C7—N3—H3	126.3	N2—C8—H8	125.1
C9—N3—H3	126.3	C8—C9—N3	106.1 (2)
C6—O1—Co	130.35 (12)	C8—C9—H9	126.9
C1—O3—Co	118.52 (12)	N3—C9—H9	126.9
O3—C1—N1	117.74 (16)		

Symmetry codes: (i) −*x*+1, *y*, −*z*+1/2.

### Hydrogen-bond geometry (Å, °)

**Table:**

*D*—H···*A*	*D*—H	H···*A*	*D*···*A*	*D*—H···*A*
N1—H1···O2^ii^	0.86	1.93	2.789 (2)	177
N3—H3···O2^iii^	0.86	2.04	2.806 (2)	148
C3—H3A···O3^iv^	0.93	2.43	3.341 (3)	168

Symmetry codes: (ii) *x*, −*y*, *z*−1/2; (iii) *x*, −*y*+1, *z*−1/2; (iv) *x*, −*y*, *z*+1/2.
